# Recent advances in understanding lipodystrophy: a focus on lipodystrophy-associated cardiovascular disease and potential effects of leptin therapy on cardiovascular function

**DOI:** 10.12688/f1000research.20150.1

**Published:** 2019-10-16

**Authors:** Thiago Bruder-Nascimento, Taylor C. Kress, Eric J. Belin de Chantemele

**Affiliations:** 1 Vascular Biology Center, Medical College of Georgia at Augusta University, Augusta, GA, USA; 2 Department of Pediatrics, Division of Endocrinology, University of Pittsburgh, Pittsburgh, PA, USA; 3 Department of Medicine, Section of Cardiology, Medical College of Georgia at Augusta University, Augusta, GA, USA

**Keywords:** lipodystrophy, cardiovascular disease, metreleptin, cardiomyopathy, hypertension

## Abstract

Lipodystrophy is a disease characterized by a partial or total absence of adipose tissue leading to severe metabolic derangements including marked insulin resistance, type 2 diabetes, hypertriglyceridemia, and steatohepatitis. Lipodystrophy is also a source of major cardiovascular disorders which, in addition to hepatic failure and infection, contribute to a significant reduction in life expectancy. Metreleptin, the synthetic analog of the adipocyte-derived hormone leptin and current therapy of choice for patients with lipodystrophy, successfully improves metabolic function. However, while leptin has been associated with hypertension, vascular diseases, and inflammation in the context of obesity, it remains unknown whether its daily administration could further impair cardiovascular function in patients with lipodystrophy. The goal of this short review is to describe the cardiovascular phenotype of patients with lipodystrophy, speculate on the etiology of the disorders, and discuss how the use of murine models of lipodystrophy could be beneficial to address the question of the contribution of leptin to lipodystrophy-associated cardiovascular disease.

## Introduction

Lipodystrophy is a group of clinically heterogeneous diseases characterized by either complete or partial absence of adipose tissue which may occur in conjunction with adipose mass redistribution and can be of either congenital or acquired origin ^1^. While inherited forms of generalized or partial lipodystrophies are exceedingly rare (1 in 10 million and 1 in 1 million, respectively) ^2^ and mainly caused by autosomal recessive mutations of the *AGPAT2*, Berardinelli-Seip congenital lipodystrophy 2 ( *BSCL2*), caveolin 1 ( *CAV1*), *PTRF* genes ^2– 12^ or lamin A/C gene ^13^, acquired forms of lipodystrophy, on the other hand, have a relatively higher prevalence with an estimated number of 100,000 patients in the United States. Autoimmune disorders and medications including highly active antiretroviral therapy in HIV-infected patients are the leading causes of acquired generalized and partial lipodystrophy ^5, 14– 16^.

Regardless of the origin of the disease, patients with lipodystrophy share common metabolic abnormalities, which include marked insulin resistance, diabetes mellitus, and hypertriglyceridemia, the severity of which is typically related to the degree of fat loss ^1^. Metabolic derangements associated with lipodystrophy develop early in life and predispose patients to pancreatitis, non-alcoholic steatohepatitis (NASH), and hepatic failure ^2, 5, 6, 17– 20^, the latter being the first cause of morbidity and mortality and of substantial reduction in lifespan (of approximately 30 years) in patients with lipodystrophy ^21^. Although less studied and described, cardiovascular disorders including hypertrophic cardiomyopathy, hypertension, and atherosclerosis are also highly prevalent in lipodystrophic patients and additional major contributors to their shortened lifespan ^21^.

A key feature of lipodystrophy is a drastic reduction in the levels of adipocyte-derived hormones including leptin, which is a major regulator of appetite, insulin sensitivity, and liver function ^22– 26^. Strong basic science and clinical evidence have demonstrated that daily supplementation with leptin in rodent models of lipodystrophy and patients with lipodystrophy restores appetite, glycemia, and hepatic and renal function ^7, 16, 18, 19, 27– 33^. Based on these key findings, metreleptin, the recombinant human leptin analog, has been adopted as the therapeutic of choice for the treatment of lipodystrophy and approved by the US Food and Drug Administration (FDA) in February 2014 for the treatment of metabolic abnormalities in patients with congenital generalized and acquired lipodystrophy ^34^. However, leptin does more than targeting the metabolic system. Leptin is a pleiotropic hormone which controls numerous organ systems and has been positively associated with hypertrophic cardiomyopathy, hypertension, and vascular inflammation in the context of obesity ^35, 36^. Whether restoring leptin levels in lipodystrophic patients with metreleptin represents a cardiovascular risk remains unclear. The goal of the present manuscript is to review the clinical and basic science literature to provide a current description of the cardiovascular diseases developed by lipodystrophy patients and rodent models of lipodystrophy and discuss the potential cardiovascular consequences of supplementing lipodystrophy patients chronically with leptin.

## Cardiovascular diseases associated with lipodystrophy

Cardiomyopathy, demonstrated by echocardiography and ECG, is a frequent finding in patients with both congenital and acquired forms of lipodystrophy, who develop similar cardiac abnormalities. A majority of patients with lipodystrophy presents hypertrophic cardiomyopathy as early as 6 months of age, as reported in a young girl with congenital generalized lipodystrophy due to seipin ( *BSCL2*) mutation ^37^. Minimal numbers of patients with lipodystrophy have features of dilated cardiomyopathy. Classically, it is believed that congenital lipodystrophy patients with underlying *BSCL2* mutation have the highest prevalence of cardiomyopathy. Up to 80% of those affected have been reported to develop left ventricular hypertrophy with frequent abnormalities on ECGs resulting from long QT syndrome and a predisposition to tachyarrhythmias, including catecholaminergic polymorphic ventricular tachycardia and sudden cardiac death. Patients with underlying *AGPAT* mutation present a lower, but still high, prevalence (53%) of left ventricular hypertrophy. Lastly, patients with acquired generalized lipodystrophy have been reported to develop cardiac hypertrophy but of a significantly milder nature ^38, 39^.

Cardiomyopathies and sudden cardiac arrest contribute to the high prevalence of death from cardiovascular causes and to the very early mortality of patients with lipodystrophy. Owing to the rarity of the disease and the paucity of patients, actual data on the cause of death in lipodystrophy patients remain scarce. Nevertheless, a recent study in 20 congenital lipodystrophy patients with *BSCL2* mutation reported a mean age of death of 27 years old, with death from cardiovascular causes representing the third cause of death after hepatic failure and respiratory infection ^16, 40, 41^.

The underlying etiology of the cardiac abnormalities in lipodystrophy remain unclear. Severe insulin resistance and hyperlipidemia, which are characteristic of lipodystrophy patients, may provide the context for the development of hypertrophic cardiomyopathy. However, hypertrophic cardiomyopathy is more frequently seen in patients with *BSCL2* mutation, who have overall milder metabolic abnormalities (including lower triglyceride levels and glycated hemoglobin) than in the AGPAT or acquired lipodystrophy groups ^38^. Hypertension, another major contributor to cardiomyopathy, affects between 30 and 50% of patients with lipodystrophy ^42, 43^. However, whether patients with *BSCL2* mutation who have the highest prevalence of cardiomyopathy are also more prone to hypertension remains unknown. One can hope that future clinical studies investigating the effects of metreleptin on cardiomyopathy will help address the question of the respective contribution of insulin resistance and hyperlipidemia, as well as hypertension, to lipodystrophy-associated cardiomyopathy. Indeed, metreleptin, the human recombinant leptin analog recently approved for the treatment of metabolic disorders associated with lipodystrophy, has proven to be efficacious at restoring insulin sensitivity and lipids levels ^7^ but failed to restore blood pressure in patients with lipodystrophy ^42^. An improved cardiac function with metreleptin would support a role for metabolic disorders in lipodystrophy-associated cardiomyopathy. Experimental studies in animal models of lipodystrophy represent an additional avenue for investigation of the underlying mechanisms.

Dyslipidemia and diabetes are leading causes of vascular disease and atherosclerosis. However, despite high prevalence of marked lipidemia and diabetes, only a few cases of atherosclerosis have been reported in lipodystrophy patients with either *BSCL2* or *AGAPT* mutations ^40^. The relatively young age of the patients at the time of the study or death may explain the low prevalence for an age-related disease. In opposition to patients with other forms of lipodystrophy, patients with familial partial lipodystrophy (FPLD) and notably females suffering from the Dunnigan-type exhibit a high prevalence of coronary artery disease most likely caused by a very severe hypertriglyceridemia ^40^. Although metreleptin treatment has proven to markedly reduce triglyceride levels in FPLD, it remains unknown whether it could reduce the incidence of atherosclerosis in these patients ^44^.

Together, these reports highlight the severity of the cardiovascular disorders developed by lipodystrophy patients and our lack of knowledge of their pathogenesis as well as stress our need for additional studies investigating their underlying mechanisms.

Table 1 summarizes the metabolic and cardiovascular alterations reported in patients with different forms of lipodystrophy.

**Table 1.** Human lipodystrophy and their characteristics.

Human disease	Genetic changes	Function of gene	Metabolic Phenotype	CV phenotype	Ref
Berardinelli- Seip congenital lipodystrophy	Mutation in *AGPAT2* and *BSCL2*	Important for lipid droplet formation and adipocyte maturation	Enlarged and fatty liver, drastic reduction in fat mass, hyperinsulinemia, hyperglycemia, and hypertriglyceridemia	Cardiac hypertrophy, LV dysfunction, calcific aortic valve, and hypertension	37, 38, 43
Mutant PPARγ	Heterozygous mutations in the ligand-binding domain of PPARγ	Adipogenesis and adipocyte differentiation	Elevated glucose and insulin	Hypertension	57, 58
Dunnigan type (FPLD2)	Mutations in *LMNA* encoding nuclear lamin A/C	Inhibits adipocyte differentiation	Insulin resistance	Hypertension and moderate LV dysfunction and dilation	38, 59, 60

AGPAT2, 1-acyl-sn-glycerol 3-phosphate O-acyltransferase 2; Bscl2, Berardinelli-Seipin congenital lipodystrophy 2; C/EBP, CCAAT-enhancer-binding proteins; CV, cardiovascular; FPLD2, familial partial lipodystrophy type 2; LV, left ventricle; PPARγ, peroxisome proliferator-activated receptor gamma; SREBP-1c, sterol regulatory element-binding protein 1.

## Cardiovascular disease in mouse models of lipodystrophy

The rare aspect of the disease, its difficult diagnosis, and its consequent paucity in patients represent major limiting factors to the study of the etiology and pathological manifestations of lipodystrophy. Fortunately, several mouse models, which reproduce the metabolic and cardiovascular abnormalities observed in humans with lipodystrophy, have been developed and employed to better analyze the origins and consequences of this rare syndrome ^45– 47^. The following section and Table 2 describe and discuss the phenotype of several of these models.

**Table 2.** Mouse models of lipodystrophy and their characteristics.

Mouse model	Genetic manipulation	Function of gene	Metabolic phenotype	CV phenotype	Ref
Caveolin 1	Global deficiency	Role in lipid droplet formation by regulating lipids and phospholipid translocation across the plasma	Elevated TG and reduced leptin plasma levels	Vascular dysfunction, right ventricular hypertrophy, cardiomyopathy, and protected from atherosclerosis ^61^	49, 62– 64
AGPAT2	Global deficiency	Catalyzes the acylation of lysophosphatidic acid to phosphatidic acid	Hyperglycemia, elevated HbA1c, hyperinsulinemia, enlarged livers, and very low adiponectin and leptin levels	Not described	65– 67
Bscl2/Seipin	Global	Important for lipid droplet formation and adipocyte maturation	Enlarged and fatty liver, drastic reduction in fat mass, plasma leptin, and adiponectin levels, hyperinsulinemia, and hyperglycemia	Cardiac hypertrophy, cardiac dysfunction, and endothelial dysfunction	45, 48, 56, 68
Bscl2/Seipin	Adipocyte-specific deficiency	Important for lipid droplet formation and adipocyte maturation	Enlarged and fatty liver, drastic reduction in fat mass, plasma leptin, and adiponectin levels, hyperinsulinemia, and hyperglycemia	Not described	48
PPARγ	Adipocyte-specific deficiency	Adipogenesis and adipocyte differentiation	Enlarged and fatty liver, reduced leptin, diabetes, and elevated TG	Not described	50, 51
Pro-renin receptor	Adipocyte-specific deficiency	Receptor for pro-renin or renin	Hyperinsulinemia, enlarged liver and pancreas, and reduced leptin plasma levels	Hypertension	52, 69
SREBP-1c	Adipocyte-specific overexpression	Lipid biosynthesis in animal cells	Hyperinsulinemia, hyperglycemia, insulin resistance, fatty liver, and reduced leptin	Not described	47, 53
A-ZIP/F-1	Adipocyte-specific deficiency	ZIP/F prevents the DNA binding of B-ZIP transcription factors of both the C/EBP and Jun families	Hyperinsulinemia, hyperglycemia, elevated TG, and reduced leptin plasma levels	Vascular dysfunction and remodeling and hypertension	54, 70, 71

AGPAT2, 1-acyl-sn-glycerol 3-phosphate O-acyltransferase 2; Bscl2, Berardinelli-Seipin congenital lipodystrophy 2; C/EBP, CCAAT-enhancer-binding proteins; CV, cardiovascular; PPARγ, peroxisome proliferator-activated receptor gamma; SREBP-1c, Sterol regulatory element-binding protein 1; TG, triglycerides.

Constitutive deletion of *BSCL2* ^48^ and *Cav1 ^–/–^* ^49^ or selective deletion of peroxisome proliferator activated receptor γ (PPARγ) ^50, 51^ and pro-renin receptor ^52^ in adipocytes has been shown to reproduce the human congenital generalized lipodystrophy syndrome in mice. Each of these mouse models exhibit a near-complete absence of adipose tissue associated with impaired glucose tolerance and hyperlipidemia. Similarly, overexpression of the sterol regulatory element-binding protein-1c (SREBP-1c) ^47, 53^ and expression of the dominant negative A-ZIP/F-1 protein ^54^ in adipose tissue reproduce well the human lipodystrophy phenotype in terms of fat mass and distribution as well as metabolic alterations. This close proximity between the metabolic phenotype of these mouse models and of human patients makes these murine models the ideal tool to investigate the etiology of cardiovascular disease in lipodystrophy.

The seipin-deficient ( *BSCL2 ^–/–^*) mouse is the model that has been the most extensively studied for its cardiovascular phenotype. Several groups have observed that *BSCL2 ^–/–^* mice, just like lipodystrophy patients ^37^, exhibit cardiac hypertrophy very early in life, as early as postnatal day 10 ^55^. Cardiac hypertrophy persists throughout adulthood and progresses to cardiomyopathy with aging ^55^. Results gathered with independent lines of *BSCL2* knockout mice concur on the structural and hemodynamic alterations induced by lipodystrophy but diverge on the pathogenesis of the cardiac phenotype. Joubert *et al*. ^56^ reported no intramyocardial lipid accumulation or lipotoxic hallmarks but detected increased myocardial glucose uptake and O-GlycNAcylated protein in *BSCL2 ^–/–^* hearts, in support of a cardiac glucose overload. Additional arguments in furtherance of an impaired cardiac glucose metabolism were provided by demonstrating that treatment with the hypoglycemic sodium glucose cotransporter 2 (SGLT2) inhibitor dapagliflozin prevented the development of hypertrophic cardiomyopathy in *BSCL2 ^–/–^* mice. Zhou *et al*. ^55^, on the other hand, identified an important link between hyperinsulinemia and organomegaly in lipodystrophic mice. They showed that activation of prohypertrophic insulin-like growth factor 1 receptor (IGF1R)-mediated PI3K/AKT signaling contributes to cardiac hypertrophy in *BSCL2 ^–/–^* mice. They also identified a unique pattern of cardiac lipid remodeling with reduced cardiac steatosis associated with adipose triglyceride lipase (ATGL) overexpression in hearts of *BSCL2 ^–/–^* mice and showed that ATGL haploinsufficiency could reverse lipodystrophy, insulin resistance, and cardiac derangements. While these two studies depart on the underlying pathological mechanisms of hypertrophic cardiomyopathy in *BSCL2 ^–/–^* mice, they strongly support a role for metabolic alterations. Interestingly, using the exact same mouse as the mouse employed by Chen *et al*. ^45^ and Zhou *et al*. ^55^, our group recently reported that lipodystrophy impairs aortic endothelium-dependent relaxation by mechanisms independent of metabolic function. Indeed, we showed that restoration of glycemia via SGLT2 inhibition failed to restore endothelial function. However, we demonstrated that the absence of adipose tissue characteristic of lipodystrophy induced a reduction in systemic leptin levels which diminished endothelial leptin signaling and caused endothelial dysfunction via an overproduction of reactive oxygen species by endothelial NADPH oxidase 1 (Nox1) ^68^. Together, these observations further highlight the complexity of the disease and suggest that metabolic alterations are not the only cause of cardiovascular disease in lipodystrophy.

The *Cav1 ^–/–^* mouse is another model that has been used to study lipodystrophy and also present cardiomyopathy ^49, 62– 64, 72^. Differently from *BSCL2 ^–/–^* mice, *Cav1 ^–/–^* mice exhibit concentric left ventricular hypertrophy and dilated right ventricular hypertrophy. The discrepancy in the cardiac phenotype between *BSCL2 ^–/–^* and *Cav1 ^–/–^* might find its origin in the etiology of the cardiomyopathy. Indeed, as described above, metabolic disorders, notably insulin resistance and hyperglycemia, appear as the primary causes of cardiomyopathy in *BSCL2 ^–/–^* mice. In opposition, in *Cav1 ^–/–^* mice, cardiomyopathy was shown to be secondary to Cav1 deletion and pulmonary hypertension. Indeed, selective restoration of Cav1 expression in endothelial cells completely rescued pulmonary hypertension and cardiac hypertrophy in *Cav1 ^–/–^* mice ^73^. Remarkably, *Cav1 ^–/–^* mice are protected from atherosclerosis, again through mechanisms independent of lipodystrophy likely involving reduction in LDL infiltration into the artery wall, increased nitric oxide production, and reduction in the expression of leukocyte adhesion molecules ^61^. Therefore, the *Cav1 ^–/–^* mouse may be less relevant to the study of lipodystrophy and its cardiovascular consequences.

A key feature of lipodystrophy is dyslipidemia, notably hyperlipidemia which, added to insulin resistance and diabetes, places patients with lipodystrophy at a high risk for atherosclerotic cardiovascular disease ^74– 79^. To investigate whether lipodystrophy predisposes to atherosclerosis, Wang *et al*. ^74^ crossed *BSCL2 ^–/–^* with low-density lipoprotein receptor ( *LDLr ^–/–^*) knockout mice, a mouse model of atherosclerosis. As observed in lipodystrophic patients ^40^, *LDLr ^–/–^ BSCL2 ^–/–^* mice present with accelerated atherosclerosis, as reflected by spontaneous plaque formation on chow diet and exacerbation of atherosclerotic lesions on atherogenic diet ^74^. The absence of adipocytes, which decreases the potential for adipose cholesterol clearance, most likely explains the extremely high rise in plasma cholesterol levels in *LDLr ^–/–^ BSCL2 ^–/–^* mice which itself predisposed lipodystrophic mice to atherosclerosis ^74^.

Lastly, mouse models of lipodystrophy, as do patients, present with hypertension ^27, 43^. Experiments conducted in transgenic A-ZIP/F-1 mice ^70, 80^ and adipose tissue pro-renin receptor-deficient mice ^52^ revealed elevated systolic blood pressure associated with hyperactivation of the renin angiotensin system (RAS). Angiotensin-converting enzyme inhibition ^81^ and angiotensin type 1 receptor blockade ^80^ restored blood pressure in these two mouse models of lipodystrophy, which further supports the contribution of RAS to the development of hypertension in mouse models of lipodystrophy and presents RAS blockade as a potential avenue for the treatment of cardiovascular disease associated with lipodystrophy. However, whether RAS overactivation is consecutive to metabolic alterations remains to be determined.

## Metreleptin and lipodystrophy-associated cardiovascular disease

Following many successful trials, the FDA has approved leptin (metreleptin) for the treatment of non-HIV-related forms of generalized lipodystrophy. Leptin replacement therapy with metreleptin has, in many cases, reversed the metabolic complications, with improvements in glucose-insulin-lipid homeostasis and regression of fatty liver disease ^7, 16, 18, 19, 27– 33^. An aspect of the treatment that remains ill-defined is whether metreleptin improves or alters cardiovascular function in lipodystrophic patients. Compelling basic science and clinical evidence indicate that excess leptin elevates blood pressure and impairs vascular function via sympatho-activation in males ^82– 86^ and aldosterone production in females ^82, 87, 88^. Therefore, concerns have been raised regarding the potential deleterious cardiovascular consequences of daily leptin injections. Recent results by Brown *et al*. partially dissipated these concerns by reporting that metreleptin did not elevate blood pressure in a relatively large population of lipodystrophic patients (107 patients) ^42^. Based on their results, the authors concluded that there was a lack of contribution of leptin to the development of hypertension and a lack of translatability of the results obtained in murine models. However, the significant improvements in glycemia, insulin resistance, and liver and renal function associated with metreleptin treatment ^18^ were not considered by the authors to reach their conclusions. Indeed, insulin resistance has been presented as a major risk factor for hypertension ^89, 90^. Therefore, the significant improvement in the metabolic profile of the lipodystrophy patients on metreleptin most certainly compensated for the indisputable effects of leptin on sympathetic tone ^91^. This may explain the lack of significant decreases in pressure in lipodystrophy patients on metreleptin. In addition, besides increasing sympathetic activity, leptin exerts vascular actions which could provide additional explanations for the lack of increase in blood pressure. Early work by the group of Lembo *et al*., and supported by others, demonstrated that leptin not only relaxes blood vessels via NO-dependent mechanisms ^92, 93^ but also controls vascular integrity by protecting vessels from neointima formation, excess endothelin 1 production, and increasing PPARγ activity ^94^. Furthermore, recent results by our group show that leptin replacement therapy restores endothelium-dependent relaxation via direct activation of endothelial leptin receptor and reduction in Nox1-derived ROS production, likely via PPARγ-dependent mechanisms, in *BSCL2 ^–/–^* mice ^57^. Taken together, these results further support the direct vascular effects of leptin and indicate that metreleptin treatment should improve vascular function in lipodystrophy patients.

Other potential concerns are the chronic effects of metreleptin on cardiac function and remodeling. Indeed, while compelling *in vitro* studies have shown that leptin promotes human and rodent cardiomyocyte hyperplasia ^95, 96^, several clinical studies have established a positive correlation between leptin and left ventricular hypertrophy after adjustment for body mass index and present leptin as an independent predictor of incident heart failure ^97^. Conversely, elegant rescue experiments involving either selective restoration of leptin receptor expression in cardiomyocytes of leptin receptor-deficient mice ( *db/db*) or restoration of leptin levels in leptin-deficient ( *ob/ob*) mice report a decreased heart mass and reduction in left ventricular wall thickness in response to leptin, supportive of the cardiac protective effects of leptin. In addition, selective cardiac leptin receptor deficiency resulted in transient left ventricular dysfunction and dramatic reduction in ejection fraction, while cardiac-specific overexpression of leptin receptors normalized cardiac triglyceride levels and diastolic function in *db/db* ^97^. All together, these data derived from murine models support a beneficial role for leptin in cardiac function and metabolism (protection from lipotoxicity) but drastically contrast with the clinical studies. This further raises the question of the potential contribution of leptin deficiency to the cardiac disorders associated with lipodystrophy and of the effects of daily metreleptin injections on the severely impaired heart function of lipodystrophic patients. Additional studies are warranted to address these concerns.

Lastly, although not tested yet, one can reasonably speculate that metreleptin exerts protective effects against atherosclerosis. While insulin resistance, diabetes, and, more specifically, hyperlipidemia are leading risk factors for atherosclerosis, compelling evidence from relatively large (66 patients) studies have demonstrated that long-term treatment with metreleptin resulted in sustained improvements in hypertriglyceridemia, glycemic control, and liver volume which led to discontinuation of insulin, oral anti-diabetics, and lipid-lowering medications in more than 25% of patients on metreleptin ^18^. Therefore, one can soundly anticipate that metreleptin will significantly reduce the risk for atherosclerosis in lipodystrophy patients through centrally orchestrated mechanisms reducing food intake but also through direct and local effects of leptin activating β-oxidation of fatty acids and preventing lipogenesis in the liver and skeletal muscles ^98^. Remarkably, another recent study reported that metreleptin treatment for 1 year reduced plasma levels of the proprotein convertase subtilisin/kexin type 9 (PCSK9), a key regulator of cholesterol metabolism, in humans with congenital lipodystrophy ^99^. This provides an additional potential mechanism whereby metreleptin might prevent atherogenesis in lipodystrophy patients. However, the hypothesis that metreleptin protects from atherosclerosis remains to be tested. Less promising and beneficial evidence from animal studies further support this need for additional studies. Indeed, while leptin deficiency has been shown to protect apolipoprotein-E-deficient mice fed an atherogenic diet from the development of atherosclerosis lesions, exogenous leptin significantly increases atherosclerotic areas in apoE-deficient mice. In addition, leptin has been shown to promote the differentiation of macrophages towards a proinflammatory phenotype ^100^, which is another major contributor to atherosclerosis. It is therefore crucial to determine whether metreleptin prevents or exacerbates atherosclerogenesis in lipodystrophy patients.

Recent studies following patients for up to 3 years have reported that metreleptin is well tolerated in patients with lipodystrophy ^18^. However, as with any other drug, metreleptin has been associated with a few side effects. Antimetreleptin antibodies with *in vitro* neutralizing activity, which could potentially reduce the drug’s efficacy or even inhibit endogenous leptin activity ^101^, have been shown to develop in most patients within 4–6 months but to decrease with continuous therapy. In addition, few patients under metreleptin treatment have been shown to develop T-cell lymphoma. However, whether metreleptin is truly a contributor requires further investigation, as patients with lipodystrophy appear to be at a higher risk for lymphoma than the general population, likely because of underlying autoimmunity ^102^. Therefore, metreleptin-associated side effects may still deserve some attention.

## Conclusion

**Figure 1.**
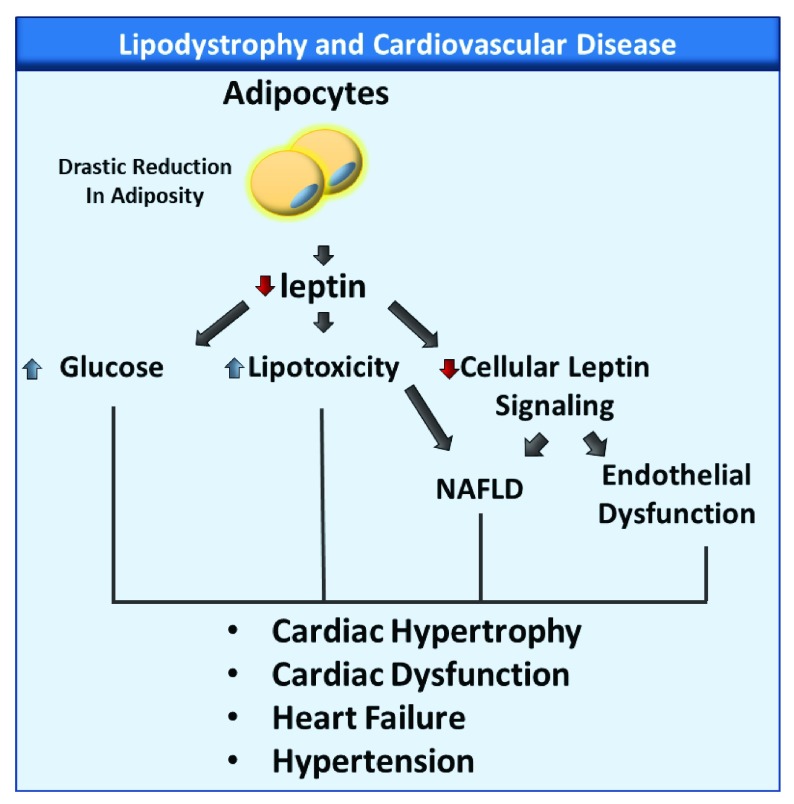
Potential Mechanisms leading to cardiovascular disease in lipodystrophy. Lipodystrophy is associated with a drastic reduction in adiposity and leptin plasma levels, which lead to hyperglycemia, lipotoxicity, and decreased cellular leptin signaling. These changes have been associated with non-alcoholic fatty liver disease (NAFLD) endothelial dysfunction, hypertension, and cardiac diseases.

In summary, while the current literature on lipodystrophy focuses mostly on the metabolic disorders associated with the syndrome, cardiovascular diseases, notably hypertension and cardiomyopathy ( Figure 1), also represent a major health concern in patients with lipodystrophy and contribute to their very early mortality. Here, we speculated that the metreleptin regimen provided to lipodystrophy patients may improve cardiovascular function through its beneficial effects on glycemia, lipidemia, and liver function. We also stressed that metreleptin may affect cardiac and vascular function through direct control of cardiomyocyte and endothelial cell function and highlighted the need for studies investigating whether metreleptin improves or impairs the function of these two types of cells. We presented several mouse models of lipodystrophy which reproduce well the metabolic and cardiovascular phenotype of patients with lipodystrophy and represent the perfect avenue to investigate the direct effects of leptin on the cardiovascular system and dissipate any potential harmful effect.
